# The Janus-faced roles of Krüppel-like factor 4 in oral squamous cell carcinoma cells

**DOI:** 10.18632/oncotarget.6256

**Published:** 2015-10-28

**Authors:** Wenwen Li, Man Liu, Ying Su, Xinying Zhou, Yao Liu, Xinyan Zhang

**Affiliations:** ^1^ Beijing Institute of Dental Research, Stomatological Hospital and School of Stomatology, Capital Medical University, Beijing, China

**Keywords:** KLF4, tumor suppressor, oncogene, oral squamous cell carcinoma

## Abstract

Krüppel-like factor 4 (KLF4) is a zinc-finger transcription factor that regulates many essential processes, including development and cell differentiation, proliferation, and apoptosis. Along with these roles in normal cells and tissues, KLF4 has important tumor suppressive and oncogenic functions in some malignancies. However, the roles of KLF4 in oral squamous cell carcinoma remain unclear. This study investigated the epigenetic alterations and possible roles of KLF4 in oral cancer carcinogenesis. Notably, KLF4 expression was significantly decreased in human oral cancer tissues compared with healthy controls, and KLF4 promoter hypermethylation contributed to the suppression of KLF4 expression. KLF4 expression was associated with tumor grade. Its expression was much lower in poorly differentiated oral cancers than in well-differentiated cancer cells. KLF4 exerted its antitumor activity *in vitro* and/or *in vivo* by inhibiting cell proliferation, cell cycle progression, cell colony formation and by inducing apoptosis. In addition, KLF4 over-expression promoted oral cancer cell migration and invasion *in vitro*. Knockdown of KLF4 promoted oral cancer cells growth and colony formation, and simultaneously inhibited cell migration and invasion. Mechanistic studies revealed that MMP-9 might contribute to KLF4-mediated cell migration and invasion. These results provide evidence that KLF4 might play Janus-faced roles in oral cancer carcinogenesis, acting both as a tumor suppressor and as an oncogene.

## INTRODUCTION

Krüppel-like factor 4 (KLF4) is a zinc finger transcriptional factor that is highly expressed in differentiated, post-mitotic cells in both gut and skin epithelium. KLF4 is also found in lung, testis, thymus, and cornea as well as in cardiac myocytes and lymphocytes [1–3]. The presence of both activation and repressor domains might allow KLF4 to exert either positive or negative transcriptional effects on its targets, depending upon the type of tissue in which it is expressed [4, 5]. As a pivotal transcriptional factor, KLF4 regulates multiple diverse essential cellular processes, including cell proliferation, differentiation, migration, and apoptosis and is involved in development and in the maintenance of normal tissue homeostasis [6, 7]. KLF4 has also been implicated in stem cell renewal and in the maintenance of pluripotency [8, 9]. Finally, KLF4 plays a key role in cancer progression and development. Mounting evidence shows that KLF4 expression and activity is altered in a large number of human cancers, including gastric [10], colorectal [11], esophageal [12], prostate [13], lung [14], bladder [15], and B-lymphocyte cancers [16]. KLF4 expression is significantly reduced in all of these tumors, consistent with its roles in cell cycle checkpoints and growth arrest. Thus, KLF4 plays an active role as a tumor suppressor. However, KLF4 was recently found to act as an oncogene in some specific cancers. For example, KLF4-transformed rat kidney epithelial cells produce tumors in mouse xenografts [17], and KLF4 expression is increased in ductal carcinoma of the breast cancer cells, in dermal squamous cell carcinomas [18, 19], and in head and neck squamous cell carcinomas [20]. Thus, KLF4 has multiple, context-dependent roles in cancer development and progression. Although much is known about KLF4, its role is not fully understood in oral cancers.

In this study, we investigated KLF4 expression and KLF4 promoter methylation in oral squamous cell carcinoma (OSCC) cell lines and in primary OSCC tissues. Viral-based over-expression and knockdown of KLF4 was utilized to investigate its functions *in vitro* and *in vivo*. Our observations indicated that KLF4 plays Janus-faced roles in OSCC, acting as both a tumor suppressor and as an oncogene.

## RESULTS

### KLF4 is down-regulated in human OSCCs

**Figure 1:**
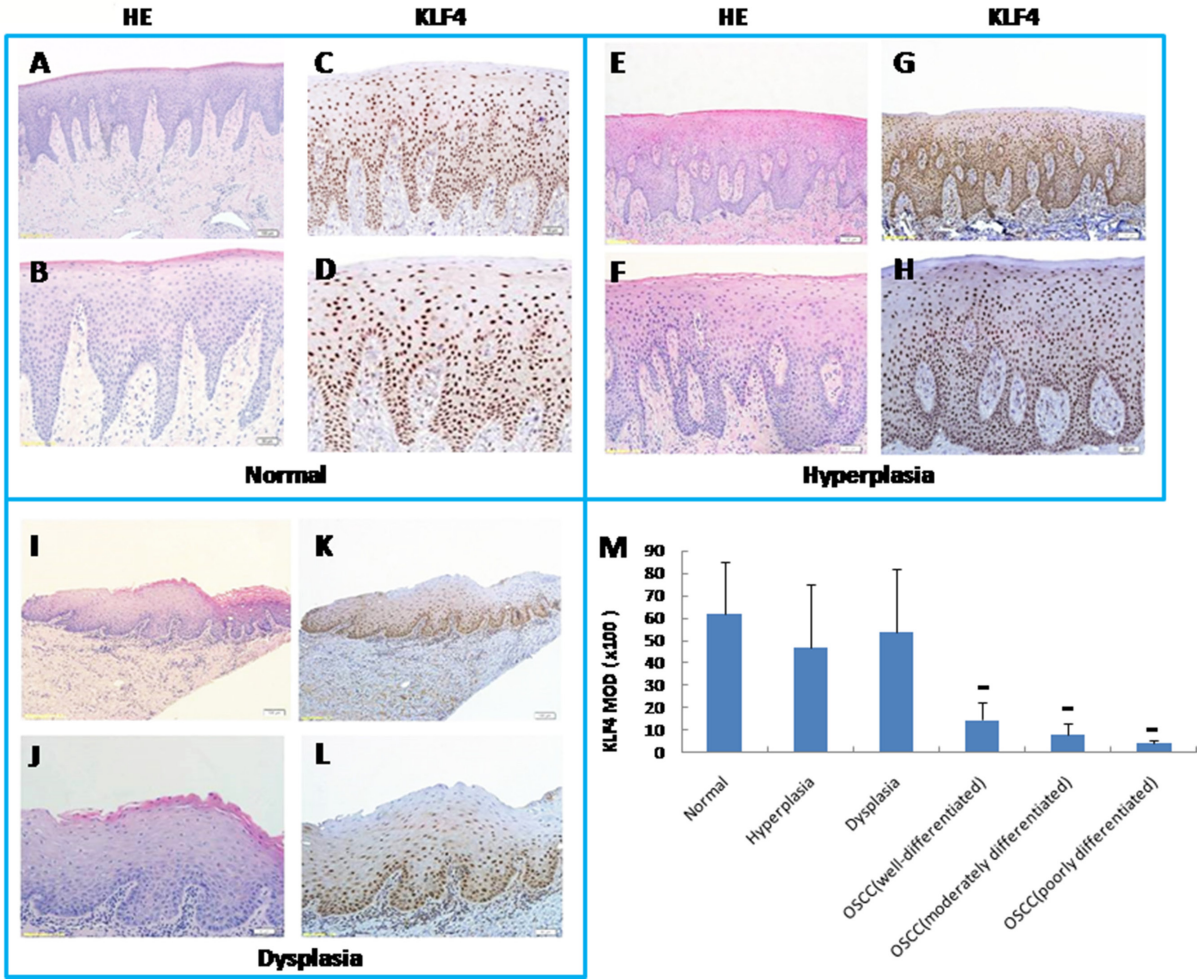
Expression of KLF4 by immunohistochemistry (IHC) in human oral mucosa and its precancerous lesions and oral squamous cell carcinomas. As well as H&E pictures of equivalent or serial sections from KLF4 pictures with low magnification and high magnification of the sample (**A** and **B**) Human healthy oral mucosa by H&E staining (A, 100x magnification; B, 200x magnification). (**C** and **D**) KLF4 expression in human healthy oral mucosa by IHC (C, 100x magnification; D, 200x magnification). (**E** and **F**) Human oral mucosa hyperplasia by H&E staining (E, 100x; F, 200x). (**G** and **H**) KLF4 expression in oral mucosa hyperplasia by IHC (G,100x; H, 200x). (**I** and **J**) Human oral mucosa dysplasia by H and E staining (I, 100x; J, 200x). (**K** and **L**) KLF4 expression in oral mucosa dysplasia by IHC (K, 100x; L, 200x). (**M**) KLF4 expression markedly decreased in OSCC samples as compared with healthy oral mucosa as displayed by MOD (mean optical density) of IHC. **P < 0.01, as compared with the healthy group.

In order to fully understand the functions of KLF4 in OSCC, we first used immunohistochemistry to determine the expression of KLF4 in human OSCC samples, in precancerous lesions (oral mucosa hyperplasia and dysplasia), and in healthy oral mucosa. KLF4 was expressed in the nuclei of epithelial cells throughout normal oral mucosa epithelium, expression was slightly decreased in hyperplasia or dysplasia, and expression was much lower in OSCC tissues (Figure 1A–1M). In OSCC tissues, KLF4 expression was associated with tumor grade and stage (Table 1). From well-differentiated OSCC to moderately differentiated OSCC, and to poorly differentiated OSCC, KLF4 staining was decreased gradually (Figure 1M, 2A–2L). As showed in Table 1, from T1 to T3 stage, KLF4 staining decreased with increasing tumor dimension, but in T4 stage (when tumors invaded adjacent structure), KLF4 expression increased a little again. But there is no statistical significance. According to clinical stage classification, although KLF4 staining got the similar pattern with TNM stage, there is still no statistical significance.

**Table 1:** KLF4 expression and clinicopathological characteristics in oral squamous cell carcinoma

Characteristic	Total (*n* = 39)	KLF4 MOD Mean ± SD	*P* Value
**Gender**			
Male	25 (64.1%)	0.0912 ± 0.06324	0.431
Female	14 (35.9%)	0.1102 ± 0.08509	
**Age (Year ± SD)**			
< 60 (50.7 ± 7.9)	18 (46.2%)	0.0960 ± 0.08356	0.876
≥ 60 (67.4 ± 7.2)	21 (53.8%)	0.0997 ± 0.06105	
**Grade**			
Well-differentiated	14 (35.9%)	0.1455 ± 0.0798	
Moderately-differentiated	19 (48.7%)	0.0802 ± 0.0552	***0.005^3^***
Poorly-differentiated	6 (15.4%)	0.0435 ± 0.0089	***0.002^3^***
**TNM Stage**			
T1 (Tumor dimension ≤ 2cm)	8 (20.5%)	0.1238 ± 0.05635	
T2 (2 cm < Tumor dimension ≤ 4 cm)	15 (38.5%)	0.1135 ± 0.08269	0.737^4^
T3 (4 cm < Tumor dimension)	5 (12.8%)	0.0512 ± 0.05024	0.076^4^
T4 (Tumor invades adjacent structure (e.g. cortical bone, deep extrinsic muscle, maxillary sinus, skin))	11 (28.2%)	0.0861 ± 0.0658	0.178^4^
**Lymph node metastasis^1^**			
N0	25 (64.1%)	0.0929 ± 0.07438	0.556
N1–2	14 (35.9%)	0.1072 ± 0.06724	
**Clinical stage^2^**			
I	7 (17.9%)	0.1275 ± 0.05982	
II	13 (33.3%)	0.1157 ± 0.08557	0.714^5^
III	16 (41.0%)	0.0650 ± 0.05895	0.051^5^
IV	3 (7.8%)	0.1287 ± 0.02732	0.98^5^

ANOVA analysis was done to determine the statistical significance of the relationship of KLF4 expression with various variables. SD, standard deviation. MOD ( mean optical density)

1 N**0**, No regional lymph node metastasis; N**1**, Metastasis in a single ipsilateral lymph node, 3 cm or less in greatest dimension; N**2**, Metastasis in a single ipsilateral lymph node, more than 3 cm but not more than 6 cm in greatest dimension; in multiple ipsilateral lymph nodes, none more than 6 cm in greatest dimension; in bilateral or contralateral lymph nodes, none more than 6 cm in greatest dimension.

2 **I**, The cancer is less than 2 centimeters in size (about 1 inch), and has not spread to lymph nodes in the area (lymph nodes are small almond shaped structures that are found throughout the body which produce and store infection-fighting cells).**II**, The cancer is more than 2 centimeters in size, but less than 4 centimeters (less than 2 inches), and has not spread to lymph nodes in the area. **III**, Either of the following: The cancer is more than 4 centimeters in size. The cancer is any size but has spread to only one lymph node on the same side of the neck as the cancer. The lymph node that contains cancer measures no more than 3 centimeters (just over one inch). **IV**, Any of the following: The cancer has spread to tissues around oral cavity. The lymph nodes in the area may or may not contain cancer. The cancer is any size and has spread to more than one lymph node on the same side of the neck as the cancer, to lymph nodes on one or both sides of the neck, or to any lymph node that measures more than 6 centimeters (over 2 inches). The cancer has spread to other parts of the body.

3 Compare with well-differentiated.

4 Compare with T1.

5 Compare with I

**Figure 2:**
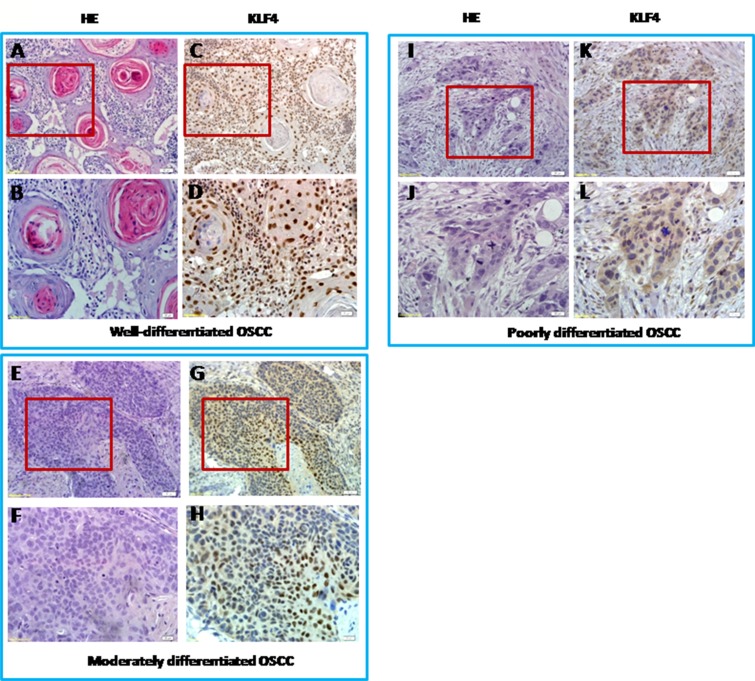
KLF4 expression in different grades of human oral squamous cell carcinomas by IHC and H&E pictures of equivalent from KLF4 pictures with low magnification and high magnification of the sample (**A** and **B)** Well-differentiated OSCC by H&E staining (A, 200x; B, 400x). (**C** and **D**) KLF4 expression in well-differentiated OSCC by IHC (C, 200x; D, 400x). (**E** and **F**) Moderately differentiated OSCC by H&E staining (E, 200x; F, 400x). (**G** and **H**) KLF4 expression in moderately differentiated OSCC by IHC (G, 200x; H, 400x). (**I** and **J**) Poorly-differentiated OSCC by H&E staining (I, 200x; J, 400x). (**K** and **L**) KLF4 expression in poorly-differentiated OSCC by IHC (K, 200x; L, 400x).

### The KLF4 promoter region is hypermethylated in OSCCs

**Figure 3:**
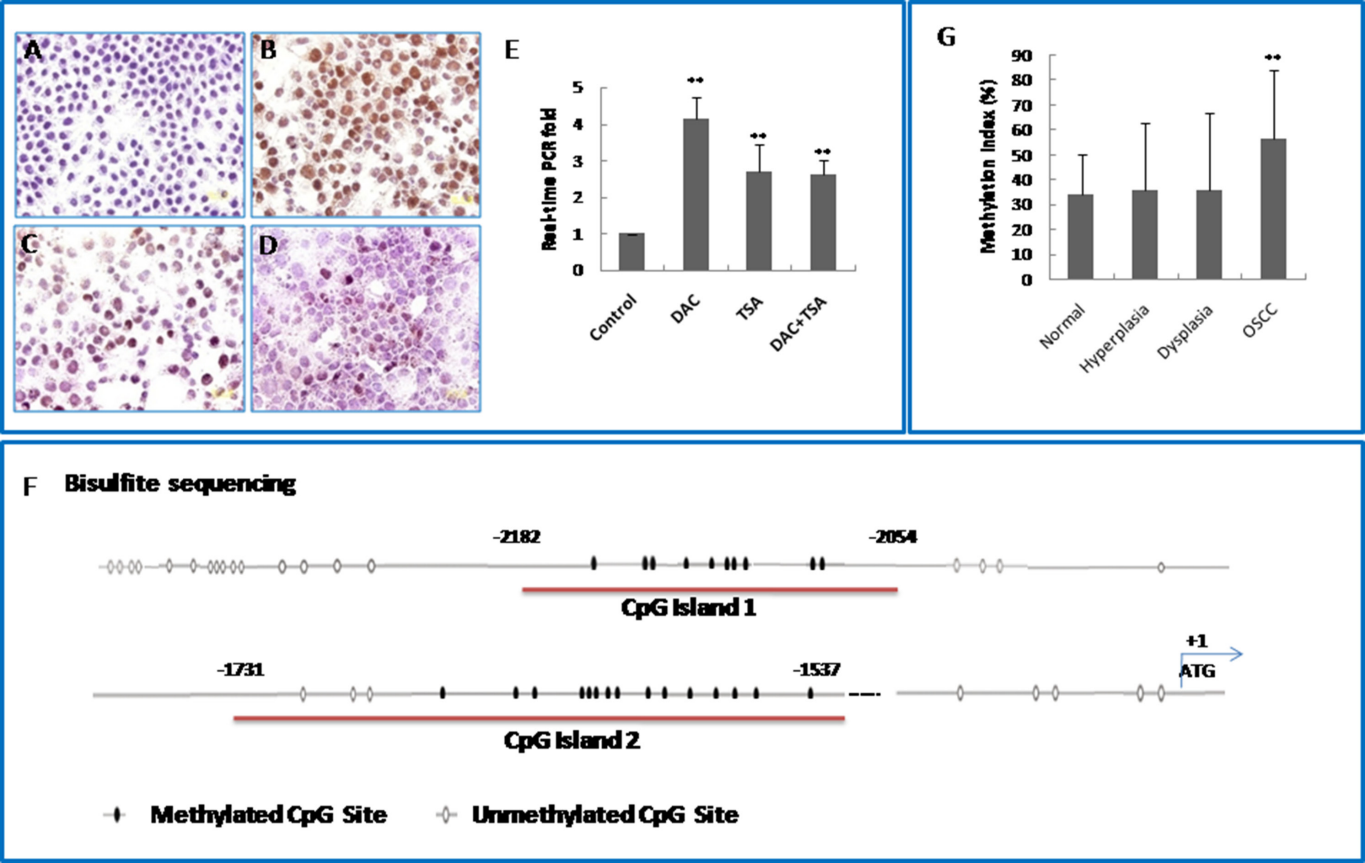
KLF4 promoter region is hypermethylated in oral squamous cell carcinomas and OSCC cell lines **(A)** Expression of KLF4 in human oral squamous cell carcinoma cell line SCC15 by immunocytochemistry. **(B)** KLF4 expression was increased markedly after treatment with 5 mM DAC for 3 days. (**C**) KLF4 expression was also increased after treatment with 300 nM TSA for 1 day. (**D**) DAC+ TSA treatment. (**E**) KLF4 expression was detected by RT real-time PCR in SCC15 cells after treatment with 5 mM DAC, 300 nM TSA alone and their combination. **P < 0.01 as compared with the control group. (**F**) A schematic representation of CpG islands found in the promoter region of the KLF4 genomic locus. Numbers indicate positions in bp relative to the transcription start site. The two CpG island regions marked in red were bisulfite sequenced. CpG sites are represented as oval, with shaded regions indicating methylation, and unshaded regions indicating no methylation. (**G**) KLF4 gene promoter methylation level was increased in human OSCC tissue as compared with health control. **P < 0.01 as compared with the normal group.

To find out whether DNA methylation and/or histone deacetylation might contribute to silencing of KLF4 gene, we used the DNA methyltransferase inhibitor DAC and the histone deacetylase inhibitor TSA to reactivate KLF4 in two OSCC cell lines, SCC15 and CAL27. KLF4 expression was detected by immunocytochemistry and by real-time PCR (Figure 3A–3E). Treatment with DAC significantly up-regulated KLF4 expression in SCC15 cells (*P* < 0.01). TSA alone also up-regulated KLF expression, but to a lesser extent than DAC alone. The combination of DAC and TSA had no synergistic effects on KLF4 up-regulation. Similar results were obtained in CAL27 cells (Supplemental Figure 1A–1F). Therefore, DNA methylation seemed to be a major silencing mechanism for KLF4 expression in human OSCC cells and histone modification might also play a role on regulation of KLF4.

The CpG methylation status of the KLF4 promoter in OSCC cells was investigated further by bisulfite sequencing. We profiled two CpG islands upstream of the KLF4 transcriptional start site, from −2182 to −2054 bp (island 1, containing 10 CpG sites), and from −1731 to −1537 (island 2, containing 15 CpG sites). The CpG sites in these two islands were hypermethylated in OSCC cells (Figure 3F). To confirm the results of the methylation sequencing, methylation-specific PCR was performed on the CpG sites of island 1 in OSCC samples and controls. The methylation level in OSCC samples (56.28%) was significantly higher than in healthy oral mucosa (34.08%) or in dysplasia (35.6%) (Figure 3G) (*P* < 0.01). Taken together, these results suggested that hypermethylation of the KLF4 promoter is involved in oral carcinogenesis.

### Over-expression of KLF4 inhibits OSCC cell growth and suppresses cell cycle progression and colony formation *in vitro*

**Figure 4:**
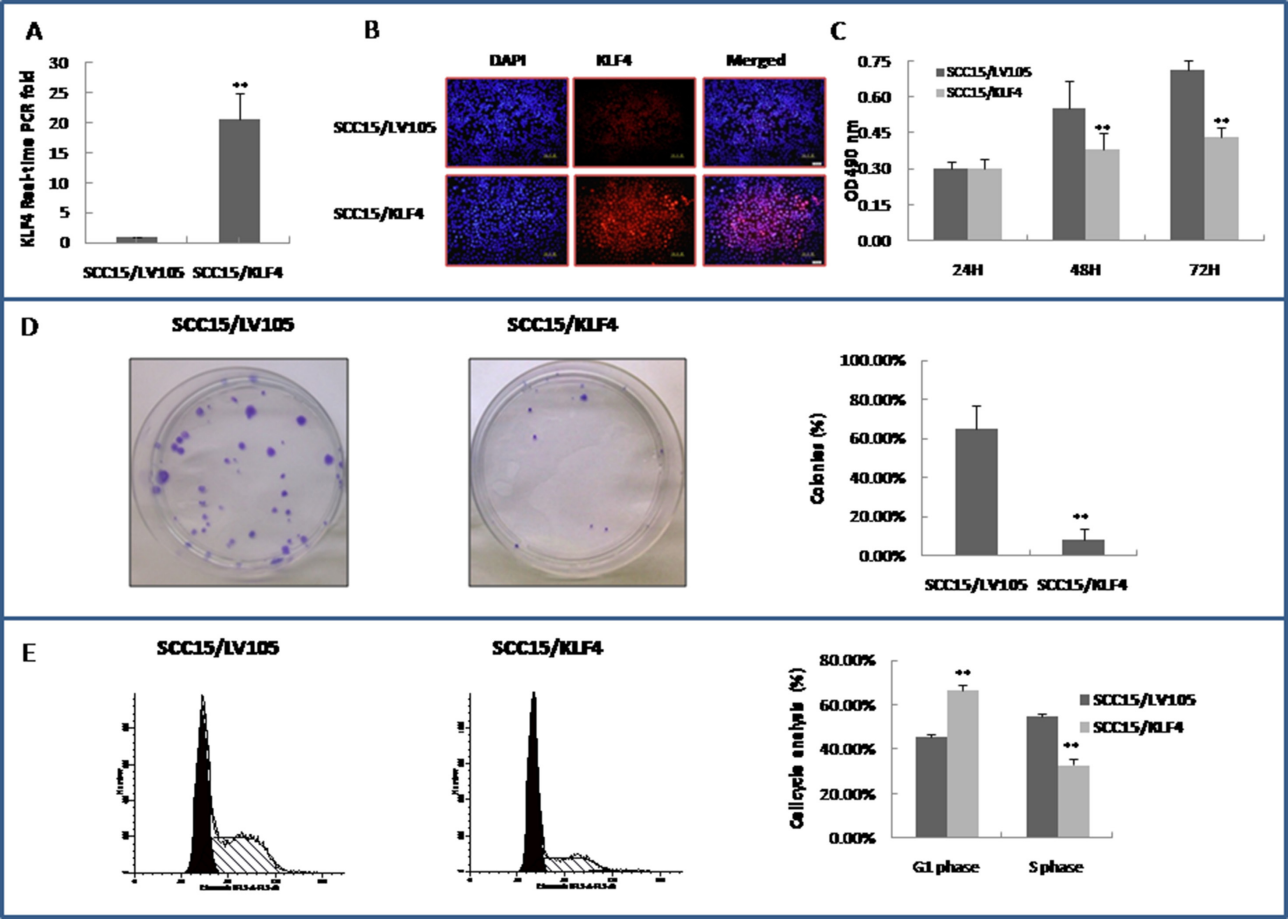
Stable transduction of KLF4 into SCC15 cells could slow down cell growth, inhibit colony formation, and cell cycle G1/S transition (**A**) Stable transduction of lentivirus LV105/KLF4 into SCC15 cells as showed by SCC15/KLF4 (SCC15/LV105 served as control) increased KLF4 expression by RT–PCR. (**B**) Stable transduction of LV105/KLF4 into SCC15 cells increased KLF4 expression and localized in the nuclei of SCC15 cells by immunocytochemistry. (**C**) SCC15/KLF4 cells grew slower than SCC15/LV105 cells, as determined by a MTT-based assay. (**D**) Colony formation assay showed that KLF4 transduction inhibited colony formation of SCC15 cells. (**E**) KLF4 transduction inhibited cell cycle G1/S transition of SCC15 cells by flow cytometry assay. *P < 0.05; **P < 0.01 as compared with the control group.

To further investigate the roles of KLF4 in oral carcinogenesis, an HIV-based lentiviral expression plasmid containing KLF4 open reading frame LV105/KLF4 was constructed and transduced into SCC15 cells. KLF4 expression was confirmed in LV105/KLF4 transduced cells and in the control by real-time PCR and by immunocytochemistry (Figure 4A and 4B). Over-expression of the KLF4 gene by the lentiviral vector slowed down SCC15 cells growth *in vitro* according to the MTT assay (Figure 4C). The colony formation assay also revealed that KLF4 over-expression markedly reduced the number and size of the colonies (Figure 4D). The cell cycle distribution was determined by flow cytometry, and over-expression of KLF4 caused a significant increase in G1 populations with concurrent declines in S populations as compared with the control (Figure 4E, *P* < 0.01). The over-expression of KLF4 experiments have also been done in another OSCC cell line CAL27 (Supplemental Figure 2A–2C). Over-expression of the KLF4 gene also slowed down CAL27 cells growth by MTT assay (Supplemental Figure 2D). But CAL27 cells lost its single colony formation ability after lentiviral infection both in the control and KLF4-transduction group. Flow cytometry assay indicated that over-expression of KLF4 in CAL27 cells inhibited cell cycle G2/M phase significantly (Supplemental Figure 2E, *P* < 0.01). These data indicated that KLF4 has a putative tumor suppressor function in oral cancer cells *in vitro*.

### Over-expression of KLF4 suppresses tumor growth in a nude mouse xenograft model by inhibiting cell proliferation and angiogenesis and by inducing apoptosis

**Figure 5:**
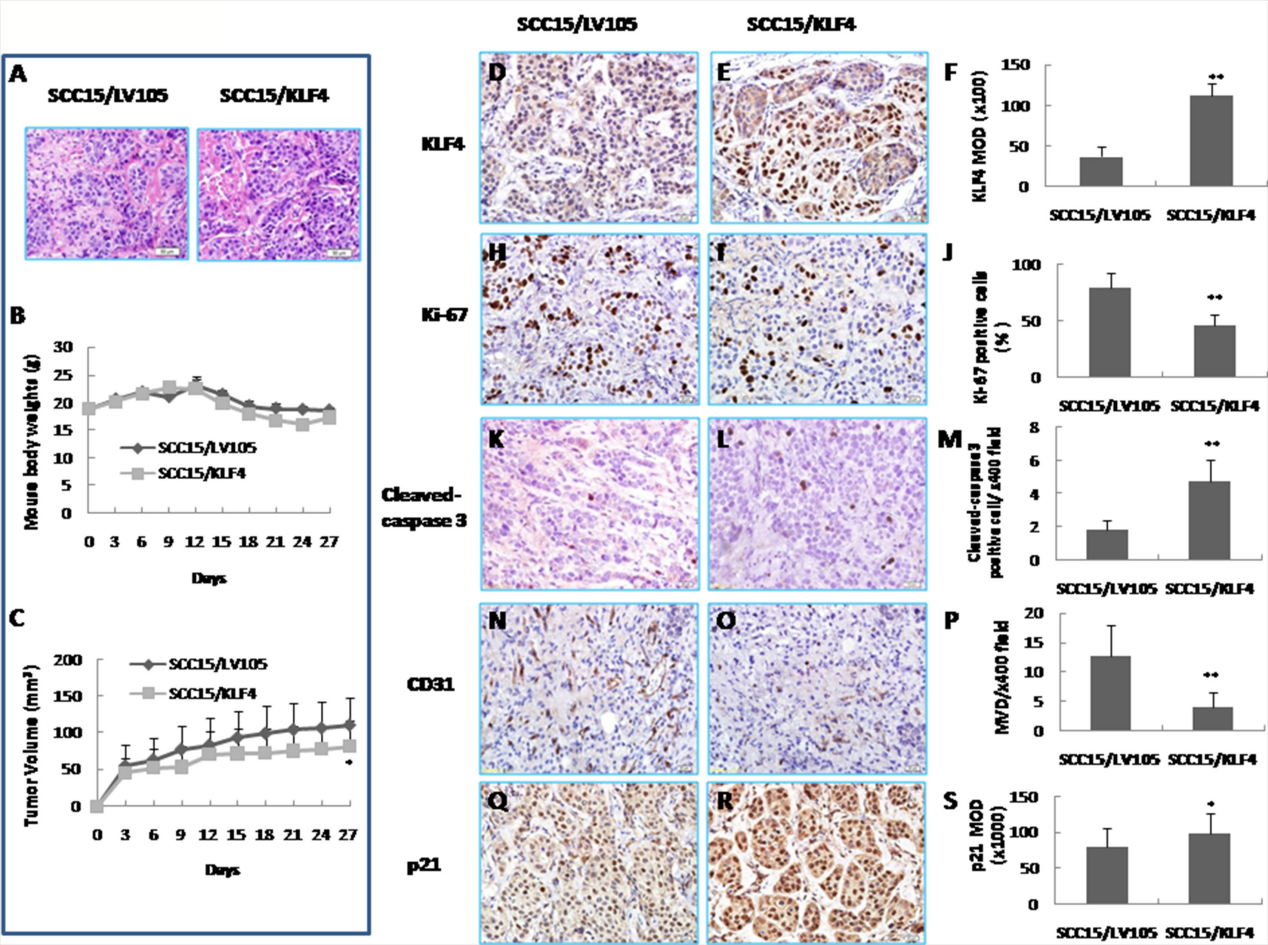
Inhibition of tumor growth *in vivo* by KLF4 transduction in a xenograft mouse model (**A**) H&E staining of xenografted tumor of nude mice. (**B**) Mouse body weights. (**C**) Tumor growth curve. *P < 0.05 as compared with the control group. (**D, E, F**) Expression of KLF4 by IHC in SCC15/LV105 and SCC15/KLF4 cells nude mice xenografted tumors. **(H, I, J**) Cell proliferation was suppressed by KLF4 transduction as showed by Ki-67 positive cells. (**K, L, M**) Apoptosis was promoted by KLF4 transduction as showed by cleaved-caspase 3 positive cells per area. (**N, O, P**) Microvessel density (MVD) of xenografted tumors was reduced by KLF4 transduction as detected by CD31 staining. (**Q, R, S**) KLF4 transduction increased p21 expression by IHC. *P < 0.05; **P < 0.01 as compared with the control group.

To confirm that KLF4 has a tumor suppressive effect *in vivo*, we established a xenograft model in BALB/c nude mice with SCC15/KLF4 cells. H&E staining showed that there is no morphological difference in the xenografted tumors between SCC15/KLF4 and the control groups (Figure 5A). KLF4 expression was confirmed in the xenograft tumors by immunohistochemistry (Figure 5D–5F). Consistent with the *in vitro* data, KLF4 gene transduction inhibited tumor growth compared to the control group as showed by a comparison of tumor volumes (Figure 5C). Immunohistochemistry analysis showed that KLF4 gene transduction reduced the percentage of Ki67-positive cells (Figure 5H–5J) and MVD (Figure 5N–5P), increased the number of cleaved caspase-3-positive cells (Figure 5K–5M), and elevated cell cycle-related gene p21 expression (Figure 5Q–5S). Thus, KLF4 exerted its antitumor activity by inhibiting tumor cell proliferation and angiogenesis and by inducing apoptosis *in vivo*. Taken together, these *in vitro* and *in vivo* data revealed that KLF4 can play a positive role by acting as a tumor suppressor in oral cancer development.

### Over-expression of KLF4 increases OSCC cell migration and invasion by elevating MMP-9

**Figure 6:**
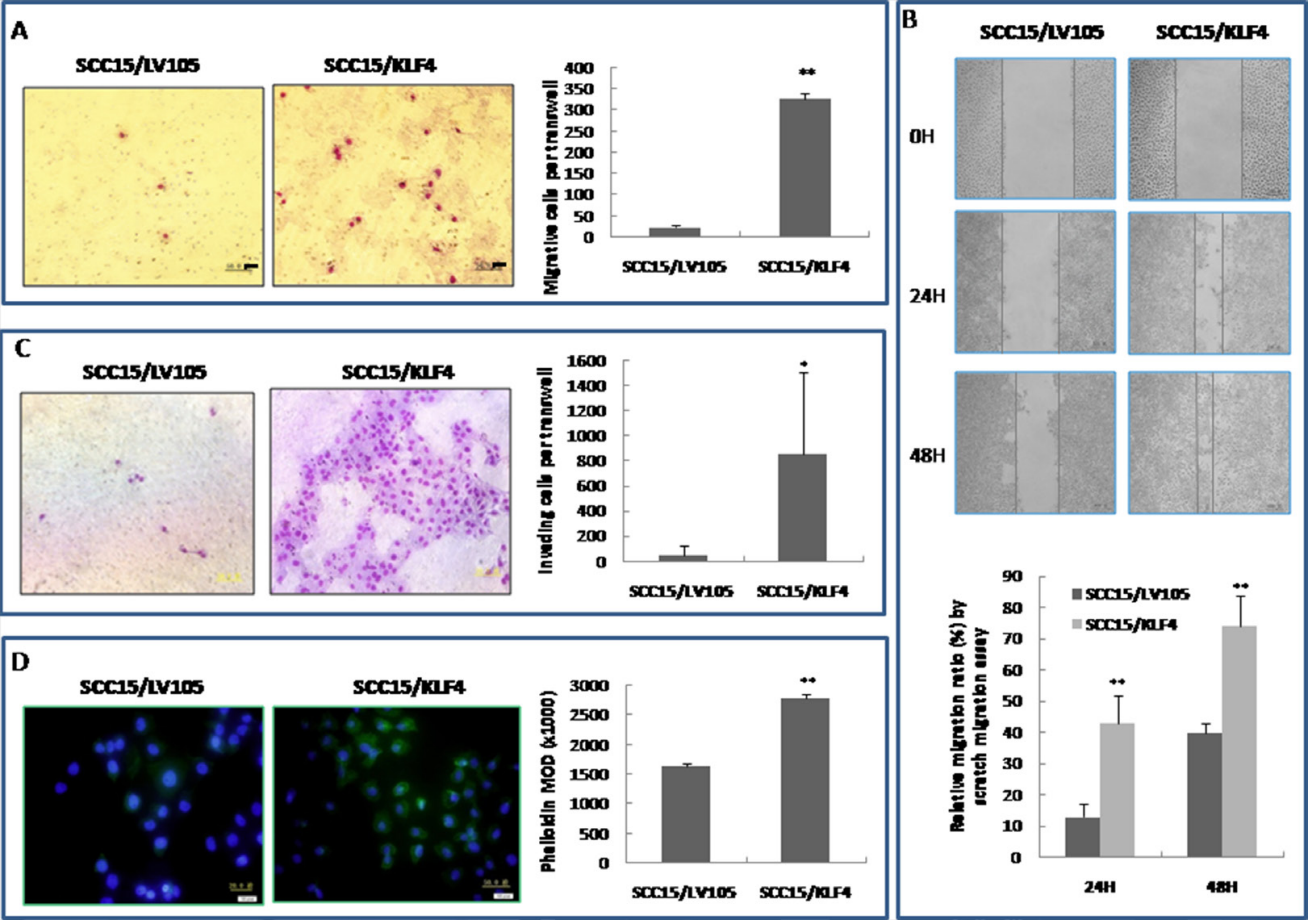
Stable transduction of KLF4 into SCC15 cells could increase cell migration and invasion ability (**A**) KLF4 transduction promoted SCC15 cell migration ability by trans-well migration assay. (**B**) KLF4 transduction promoted SCC15 cell migration ability by scratch migration assay. (**C**) KLF4 transduction promoted SCC15 cell invasive ability by trans-well invasion assay. (**D**) KLF4 transduction increased phalloidin staining, which reflects actin cytoskeleton remodeling. *P < 0.05; **P < 0.01 as compared with the control group.

The ability of SCC15 cells that were stably transduced with KLF4 to migrate and invade was assessed *in vitro* by the scratch assay and by the transwell migration and invasion assay. In contrast to a previous report that KLF4 inhibits both migration and invasion in renal cancer cells [21], over-expression of KLF4 significantly promoted cell migration in the scratch assay and transwell migration assay compared with the control cells (Figure 6A and 6B, *P* < 0.01). Over-expression of KLF4 also significantly increased cell invasion in the transwell invasion assay (Figure 6C, *P* < 0.05). Finally, FITC-phalloidin labeling of F-actin showed that actin expression was also significantly increased in SCC15 cells that were stably transduced with KLF4 (*P* < 0.01); this reflects increased actin cytoskeleton remodeling which affects cell migration (Figure 6D).

**Figure 7:**
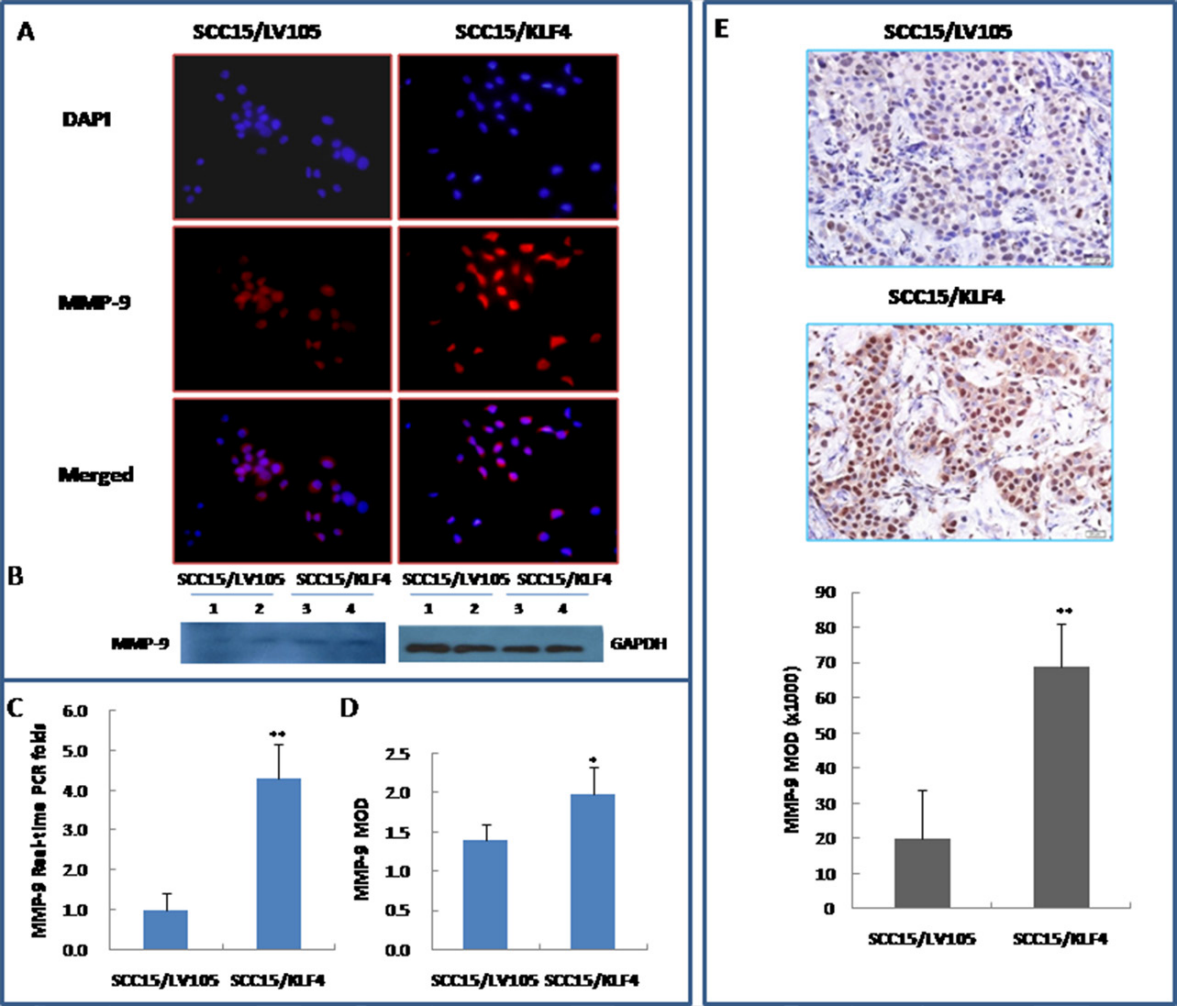
Stable transduction of KLF4 into SCC15 cells increased MMP-9 expression (**A** and **D**) KLF4 transduction increased MMP-9 expression by immunocytochemistry. (**B**) KLF4 transduction increased MMP-9 expression by western blotting. (**C**) KLF4 transduction increased MMP-9 expression by Real-time PCR detection. (**E**) KLF4 transduction increased MMP-9 expression by IHC in SCC15/KLF4 xenografted tumors. *P < 0.05; **P < 0.01 as compared with the control group.

To further investigate the mechanism by which KLF4 promotes migration and invasion, the mRNA and protein expression of MMP-9 (a key mediators of tumor cell invasion) were examined in SCC15/KLF4 cells. Expression of MMP-9 was significantly increased in SCC15/KLF4 cells by immunocytochemistry (Figure 7A and 7D), western blotting (Figure 7B) and real-time PCR (Figure 7C). Even in the SCC15/KLF4 cell xenograft tumors, MMP-9 expression was also increased as compared with the control group as determined by immunohistochemistry (Figure 7E).

Cell migration and invasion assays and MMP-9 expression detection have also been conducted in CAL27/KLF4 and CAL27/LV105 transduction cells and similar results were obtained, as showed in Supplemental Figure 3A–3D, and Supplemental Figure 4A–4C. Taken together, these findings indicate that KLF4 expression promotes oral cancer cell migration and invasion, which is associated with MMP-9 expression.

### Knockdown of KLF4 promoted OSCC cell growth and increased colony formation *in vitro*

**Figure 8:**
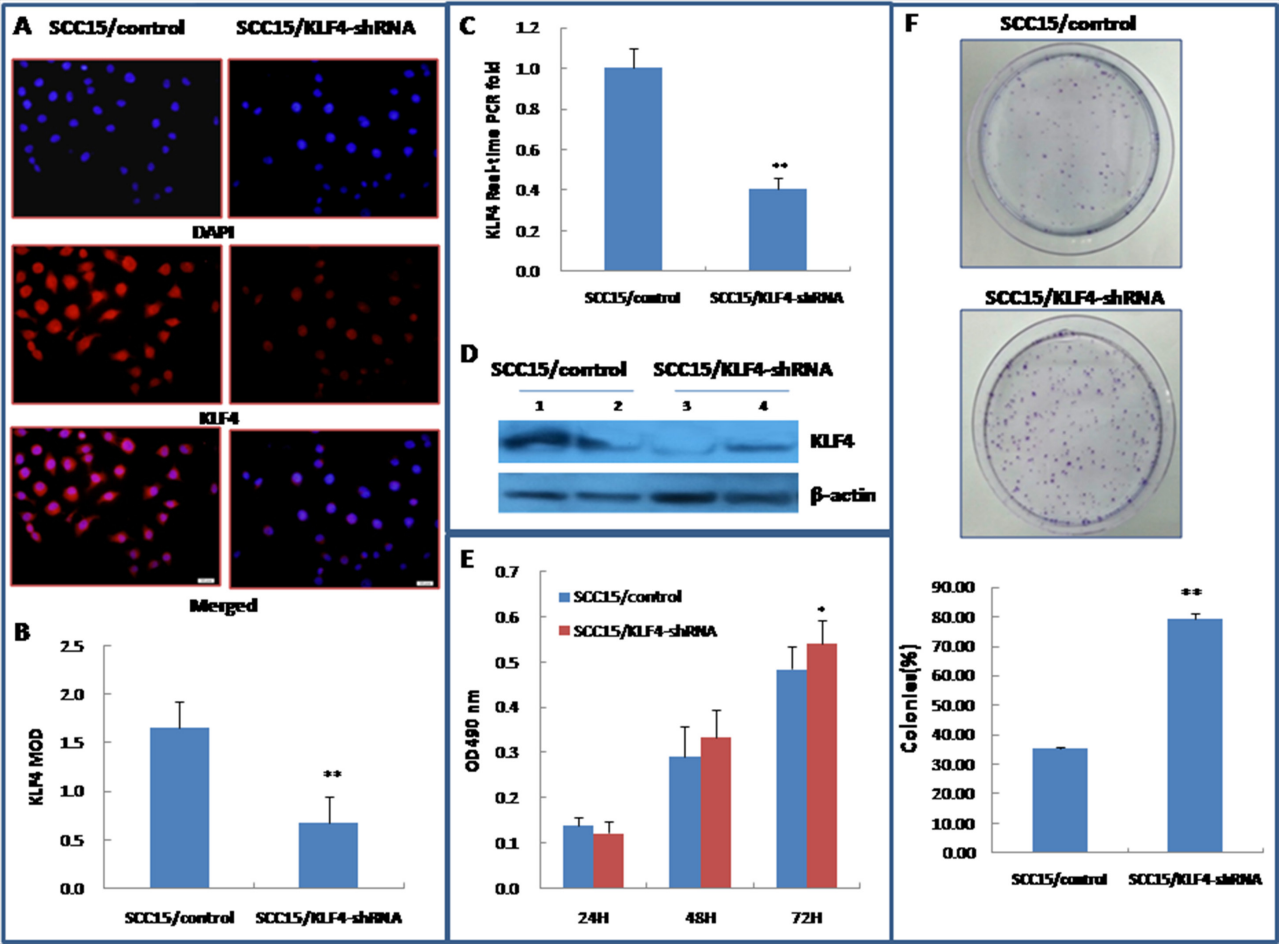
Stable transduction of KLF4-shRNA lentivirus into SCC15 cells could increase cell growth and colony formation (**A∼D**) Stable transduction of lentivirus LV3/KLF4-shRNA into SCC15 cells as showed by SCC15/KLF4-shRNA (SCC15/control served as control) decreased KLF4 expression by immunocytochemistry (A&B), by RT–PCR(C), and by western blotting (D). (**E**) Stable transduction of LV3/KLF4-shRNA increased SCC15 cell growth, as determined by a MTT-based assay. (**F**) KLF4-shRNA transduction increased colony formation of SCC15 cells. *P < 0.05; **P < 0.01 as compared with the control group.

To further confirm the Janus-faced roles in OSCC cells, a HIV-based lentiviral expression plasmid containing KLF4-shRNA was constructed and transduced into SCC15 cells. KLF4 expression was confirmed in LV3/KLF4-shRNA transduced cells and in the control by immunocytochemistry, by real-time PCR and western blotting (Figure 8A–8D). Knockdown of the KLF4 gene by the lentiviral vector promoted SCC15 cells growth *in vitro* according to the MTT assay (Figure 8E). The colony formation assay also revealed that KLF4 knockdown markedly increased the number of the colonies (Figure 8F). The knockdown of KLF4 experiments have also been done in another OSCC cell line CAL27 (Supplemental Figure 5A–5B). Because lentiviral infection seriously influenced CAL27 cells growth both in the control and KLF4-shRNA-transduction group, there is no significant growth difference between the two groups by MTT assay (Supplemental Figure 5C). And CAL27 cells also lost its single colony formation ability after lentiviral infection both in the control and KLF4-shRNA-transduction group. These data further confirmed cell growth inhibitory effect of KLF4 in oral cancer cells *in vitro*.

### Knockdown of KLF4 inhibited OSCC cell migration and invasion by reducing MMP-9 expression *in vitro*

**Figure 9:**
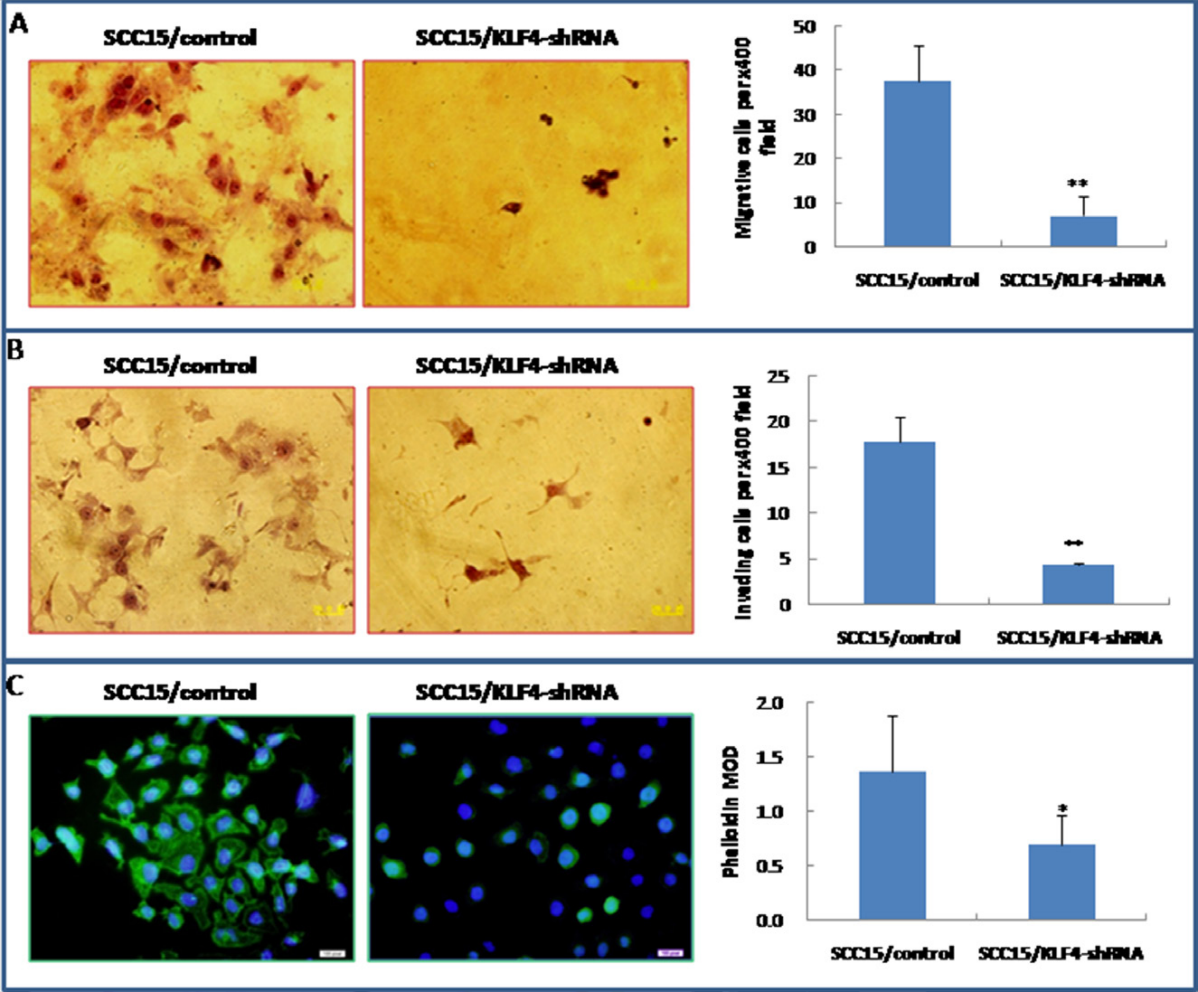
Stable transduction of KLF4-shRNA into SCC15 cells could decrease cell migration and invasion ability (**A**) KLF4-shRNA transduction inhibited SCC15 cell migration ability by trans-well migration assay. (**B**) KLF4-shRNA transduction inhibited SCC15 cell invasive ability by trans-well invasion assay. (**C**) KLF4-shRNA transduction decreased phalloidin staining. *P < 0.05; **P < 0.01 as compared with the control group.

**Figure 10:**
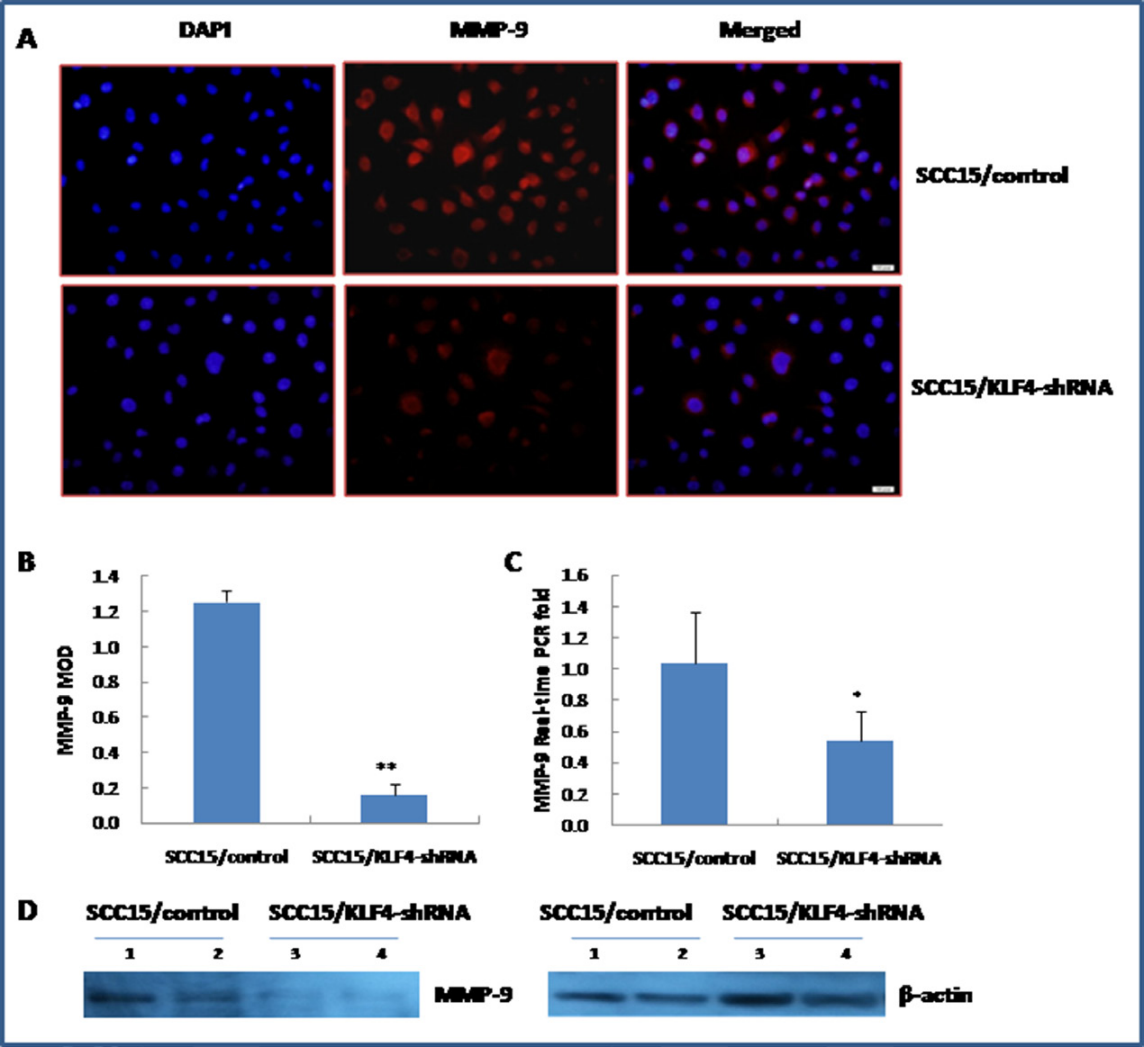
Stable transduction of KLF4-shRNA into SCC15 cells decreased MMP-9 expression (**A** and **B**) KLF4-shRNA transduction decreased MMP-9 expression by immunocytochemistry. (**C**) KLF4-shRNA transduction decreased MMP-9 expression by Real-time PCR detection. (**D**) KLF4-shRNA transduction decreased MMP-9 expression by western blotting. *P < 0.05; **P < 0.01 as compared with the control group.

Transwell migration and invasion assay indicated that knockdown of KLF4 gene significantly inhibited SCC15 cell migration and invasive ability (Figure 9A and 9B, *P* < 0.01). FITC-phalloidin labeling of F-actin was also significantly decreased in SCC15 cells that were stably transduced with KLF4-shRNA (Figure 9C, *P* < 0.01). Expression of MMP-9 was significantly decreased in SCC15/KLF4-shRNA cells by immunocytochemistry (Figure 10A and 10B), real-time PCR (Figure 10C) and western blotting (Figure 10D).

Cell migration and invasion assays and MMP-9 expression detection have also been conducted in CAL27/KLF4-shRNA and the control transduction cells and similar results were obtained, as showed in Supplemental Figure 6A–6C, and Supplemental Figure 7A–7C. These data further confirmed cell migration and invasion promotion ability of KLF4 in oral cancer cells *in vitro*.

Taken together, all of the knockdown experiments further confirmed the Janus-face roles of KLF4 in OSCC cells.

## DISCUSSION

The function of KLF4 is context-dependent, during cancer development and progression, and in different cancer types. Deletion of the KLF4 gene in a mouse model leads to abnormal cell differentiation, increased cellular proliferation, and the formation of intestinal adenomas in the colon and gastric epithelia [22]. KLF4 acts as an inhibitor of cell-cycle progression by transcriptional activation of p21 or p27 and by repression of CCNB1 and CCND1 [21, 23]. These observations provide compelling evidence that KLF4 has putative tumor suppressor functions in a variety of malignancies.

Consistent with previous studies, this study found that KLF4 was mainly expressed in the nuclei of epithelial cells throughout the entire epithelial layer of human oral mucosa and that its expression was significantly decreased in OSCC tissues. In OSCC tissues, KLF4 expression was associated with tumor classification and tumor size. KLF4 expression was greatly down-regulated in poorly differentiated OSCCs as compared to well-differentiated OSCCs. Further mechanistic studies confirmed that KLF4 functions as a tumor suppressor in OSCC development.

In cultured and nude mouse xenografts of OSCC cells, KLF4 over-expression inhibited cell proliferation and colony formation, promoted cell cycle arrest *in vitro*, and inhibited tumor growth, suppressed angiogenesis, induced apoptosis, and transcriptionally activated p21 *in vivo*. And KLF4-shRNA knockdown could reverse the growth inhibitory effect in OSCC cells. Moreover, KLF4 promoter methylation was also observed in oral cancer cell lines and oral cancer tissues, and this methylation played an important role in KLF4 down-regulation. In addition, histone modification might also play a role on regulation of KLF4. These *in vitro* and *in vivo* results suggest that KLF4 plays a tumor suppressive role in oral cancer development.

Things are not as simple as we thought previously. KLF4 acts not only as a tumor suppressor; it also has a dark side in that it acts as an oncogene in OSCC progression. Forced over-expression of KLF4 accelerated OSCC cells migration and invasion, and KLF-shRNA knockdown reduced OSCC cells migration and invasion ability *in vitro*. Mechanistically, MMP-9, a key mediator of tumor cell invasion, might involve in the KLF4-mediated migration and invasion. Lots of reports demonstrated KLF4 inhibited epithelial-mesenchymal transition (EMT) by activating E-cadherin [24, 25]. On the contrary to the previous reports, E-cadherin were down-regulated (Supplemental Figure 8) and some EMT transcription factors were up-regulated in KLF4 transduction cells by real-time PCR. But in KLF4 knockdown experiments, these results could not be reversed, so we did not show the data here.

KLF4 has been reported to be an oncogene mainly in breast cancer. KLF4 is highly expressed in more than 70% of breast cancers and is especially enriched in cancer stem cell (CSC)-like cells from both mouse primary mammary tumors and in human breast cancer cell lines. Increased nuclear staining of KLF4 is associated with a more aggressive phenotype [26], while KLF4 knockdown suppresses cell migration and invasion in MCF-7 and MDA-MB-231 cells [27]. In addition, recent evidence demonstrates that KLF4 plays a role in cellular migration and invasion during embryogenesis [28].

Despite the conflicting reporting that indicate that KLF4 plays both tumor suppressing and oncogenic roles in epithelial cancer biology, its function still need to be clarified in the future. KLF4 these context-dependent functions might be partly mediated by molecular switches such as p53, p21, or SIN3 transcription regulator homologue A. The exact mechanism by which KLF4 switches between these two opposing modes remains largely unknown. Structurally, KLF4 has both activation and repression domains, allowing it to bind to different co-activators, co-repressors, and modifiers, resulting in diverse functionality and specificity. Furthermore, alternative splicing or post-translation modifications of KLF4 can lead to additional alterations in protein structure, resulting in additional functional diversity [29].

Although the *in vitro* and *in vivo* data revealed the Janus-faced character of KLF4, which acted both as a tumor suppressor and as an oncogene in oral cancer development and progression. There are still much limitations in this study. For example, this study only focused on tumor cells, did not take account of immune system influence in tumor metastasis. It is reported that in some cancers, immunosurveillance plays an integral role in tumor initiation, growth and metastasis. Myeloid-derived suppressor cells (MDSC) and Tregs play essential roles in immunosuppression and tumor progression. MDSC recruitment expansion is promoted when tumor and stromal cells release a variety of cytokines and other soluble factors including MMPs, VEGF, and TGF-β [30]. In addition, KLF4 plays an important role in T cell development and differentiation by controlling T cell number and its function on tumor microenvironment is still unknown.

Although much is known about KLF4, the exact molecular mechanisms by which KLF4 works as a transcriptional activator, repressor, tumor suppressor, and oncogene, is not fully elucidated. Identification of KLF4's binding partners, effectors, and downstream targets might help explain its behavior in different tissues. Further studies are needed to clarify these mechanisms.

## MATERIALS AND METHODS

### KLF4 expression in human OSCCs

Using immunohistochemistry, KLF4 expression was assessed in human healthy oral mucosa, in oral mucosa hyperplasia and dysplasia, and in OSCC tissues. Human tissue sections were obtained from Beijing Stomatological Hospital, Capital Medical University after gaining approval from the ethics committee. All of the specimens were assessed by H&E staining and examined microscopically by pathologists. Oral mucosa dysplasia and OSCC specimens were diagnosed according to the World Health Organization classification system (2006) of Head and Neck Tumors; OSCC was staged according to the TNM classification system (2002) of the Union for International Cancer Control (UICC). Healthy oral mucosa tissues were from gingival mucosa and obtained from periodontal surgery. Immunohistochemistry was performed according to the instructions supplied by Abcam. The primary antibodies for KLF4 (1:300) were obtained from Proteintech (Chicago, IL).

### Cell lines and treatments

The OSCC cell lines CAL27 and SCC15 were obtained from the American Type Culture Collection (Manassas, VA). The cell lines were routinely cultured in DMEM:F12 or DMEM medium (Invitrogen Life Science, Carlsbad, CA) supplemented with 10% FBS, 100U/ml penicillin, and 100 μg/ml streptomycin. All cultured cells were maintained at 37°C in a 5% CO_2_-humidified atmosphere. All cell culture experiments were performed in triplicate to ensure reproducibility. For 5-aza-2-deoxycytidine (DAC) treatment, 70% confluent cells were treated with 5 mM DAC for 72 h. Some cells were treated with tricostatin A (TSA, 300 nM) for 24 h. For the combination experiment, cells were treated with DAC for 72 h and then with TSA for an additional 24 h.

### Real-time reverse transcription PCR

Total RNA was isolated from cells with Trizol, and reverse transcription was performed with the PrimerScript RT Master Mix (Invitrogen). The qPCR primer sets for KLF4 and MMP-9 were provided by GeneCopoeia. The ΔΔCt method was used for quantification, and GAPDH was used as an endogenous control.

### Bisulfite sequencing and methylation-specific PCR

Genomic DNA was extracted from OSCC cell lines using the DNeasy Blood & Tissue Kit (Qiagen, Valencia, CA), according to the manufacturer's instructions. Genomic DNA was bisulfite-modified with the EZ DNA Methylation-Direct Kit (Zymo Research Co., Orange, CA), and the modified DNA was amplified and examined by electrophoresis in a 1.5% agarose gel to confirm that a single band had been obtained. It was then sequenced with the 3730 xl system (Applied Biosystems, Warrington, United Kingdom). The bisulfite sequencing results were analyzed with BISMA, an online software tool.

The methylation status of KLF4 was further validated in healthy oral mucosa, hyperplasia, dysplasia and OSCC samples. Bisulfite conversion was carried out using the Methylamp DNA Modification Kit (Epigentek, Farmingdale, NY). Methylation analysis was performed using a fluorescence-based, real-time PCR assay with the following conditions: 95°C for 10 min, 35 cycles (95°C 15 s, 60°C 60 s), then 72°C for 7 min.

### Lentivirus production and transduction

The KLF4 Lentiviral ORF Expression Clone was obtained from GeneCopoeia Inc. Recombinant lentiviral particles were generated by co-transfecting an HIV-based lentiviral expression plasmid, pReceiver-LV105/KLF4, with the Lenti-Pac HIV Expression Packaging Kit into 293Ta lentiviral packaging cells. Lentiviruses containing the KLF4 gene were transduced into the target SCC15 and CAL27 cells with Envirus-LV (Engreen Biosystem, Co, Ltd), and KLF4 stably transduced cells were selected and expanded by puromycin treatment. The lentiviral transfer vector that expresses the eGFP protein served as the control. The KLF4-shRNA Lentiviral Clone and control virus was obtained from GenePharma Inc.

### Proliferation and cell cycle assays

Cell growth was assessed using the MTT assay. SCC15/LV105 (control) and SCC15/KLF4 stably transduced cells (5 × 10^4^/ml) were plated in 96-well plates and incubated for 24 h, 48 h, and 72 h. Cell survival was assessed by adding 20 μL of MTT solution (5 mg/ml) to each well and incubating at 37°C for 4 h. The reaction products were dissolved by adding 150 μL DMSO, and the plates were read using a microplate reader at a wavelength of 490 nm. To determine the cell-cycle distribution, 1 × 10^6^ cells were fixed and stained with propidium iodide (PI; Sigma-Aldrich) in cold 70% ethanol. The DNA content was determined by flow cytometry, and the data were analyzed using the ModFit cell-cycle analysis software (Verity Software House).

### Tumor cell colony formation assay

A cell suspension was prepared and added to a culture dish at a density of 50 cells/cm^2^. After 10 days of culture, the cells were fixed with methanol and stained with Giemsa staining solution. The colonies were counted and photographed using a microscope and 40x magnification.

### Cell migration and invasion assays

For the scratch assay, SCC15/KLF4 cells and control cells were grown in 24-well plates. After the cells had reached confluence, we inflicted a uniform wound in a straight line in each well using a pipette tip and then washed the wounded cells with PBS to remove all cellular debris. The cells were cultured in serum-free medium at 37°C in 5% CO_2_. We evaluated the closure or filling in of the wounds at 24 h and 48 h using microscopy with 40x magnification.

For the transwell migration assay, stably transduced SCC15/LV105 and SCC15/KLF4 cells (2 × 10^5^) in serum-free medium were placed into the upper chamber of a culture-plate insert (8-μm pore size; BD Bioscience, Franklin Lakes, NJ). Medium containing 10% fetal bovine serum was added to the lower chamber. After 24 h of incubation, the cells remaining on the upper membrane were removed using cotton balls, and the cells that had passed through the membrane were fixed with methanol and stained with Giemsa. The number of cells was counted under a microscope.

To evaluate cell invasion, transwell membranes were coated with Matrigel prior to plating infected cells. The Matrigel serves as a basement membrane barrier that cells have to destroy in order to invade the lower chamber. After 24 hours, the cells remaining on the upper membrane were removed with cotton balls, and Giemsa staining and cell counting were performed as described above.

### Tumor development in a nude mouse xenograft model

Six-week-old BALB/c nude mice were divided randomly into 2 groups: group A received control SCC15/LV105 cells, and group B received SCC15/KLF4 cells. Tumor cells (100 μL, 5 × 10^6^ cells) were mixed with 100 μL of Matrigel, and the mixture was inoculated into mice. Mouse body weight and tumor volume were measured twice a week, and the mice were sacrificed 4 weeks later. Tumors were removed, their volume was measured, and the tumors were fixed in neutral formalin for histopathological examination. All experimental procedures were in accordance with the institutional guidelines of the Animal Care and Welfare Committee of the School of Stomatology, Capital Medical University.

### Histopathology and immunohistochemistry

Tumor tissues were embedded in paraffin wax. Sections (4-μm thick) were stained with H&E and assessed microscopically. Immunohistochemistry was performed according to the instructions supplied by Abcam. Microvessel density (MVD) was calculated by counting the number of CD31-positive microvessels per each x200 field.

### Western blotting

For Western blotting, cell lysates were loaded on 4–20% acrylamide gels, and the proteins were separated by electrophoresis and transferred onto a nitrocellulose membrane. The blots were probed with primary antibody followed by horseradish peroxidase-conjugated secondary antibody and detected by chemoluminescence using ECL reagent (Millipore Corporation, MA). GAPDH and β-actin served as loading controls.

### Statistical analysis

Data from all experiments were expressed as mean ± SD. Statistical differences were determined by the ANOVA test for comparisons between means. *P* < 0.05 was considered significantly.

## SUPPLEMENTARY MATERIAL FIGURES



## Abbreviations

DAC: 5-aza-2′-deoxycytidine
ECAD: E-cadherin
EMT: epithelial-mesenchymal transition
KLF4: Krüppel-like factor 4
MVD: microvessel density
OSCC: oral squamous cell carcinoma
PI: propidium iodide
TSA: trichostatin A.
