# Supplementary material for: The Janus-faced roles of Krüppel-like factor 4 in oral squamous cell carcinoma cells
Source: Oncotarget. 2015 Oct 28;6(42):44480–94. doi: 10.18632/oncotarget.6256 (PMC4792570; doi:10.18632/oncotarget.6256)
Supplement: Supplementary file 1 [file oncotarget-06-44480-s001.pdf]

The Janus-faced roles of Krüppel-like factor 4 in oral squamous cell carcinoma cells

Supplementary Materials

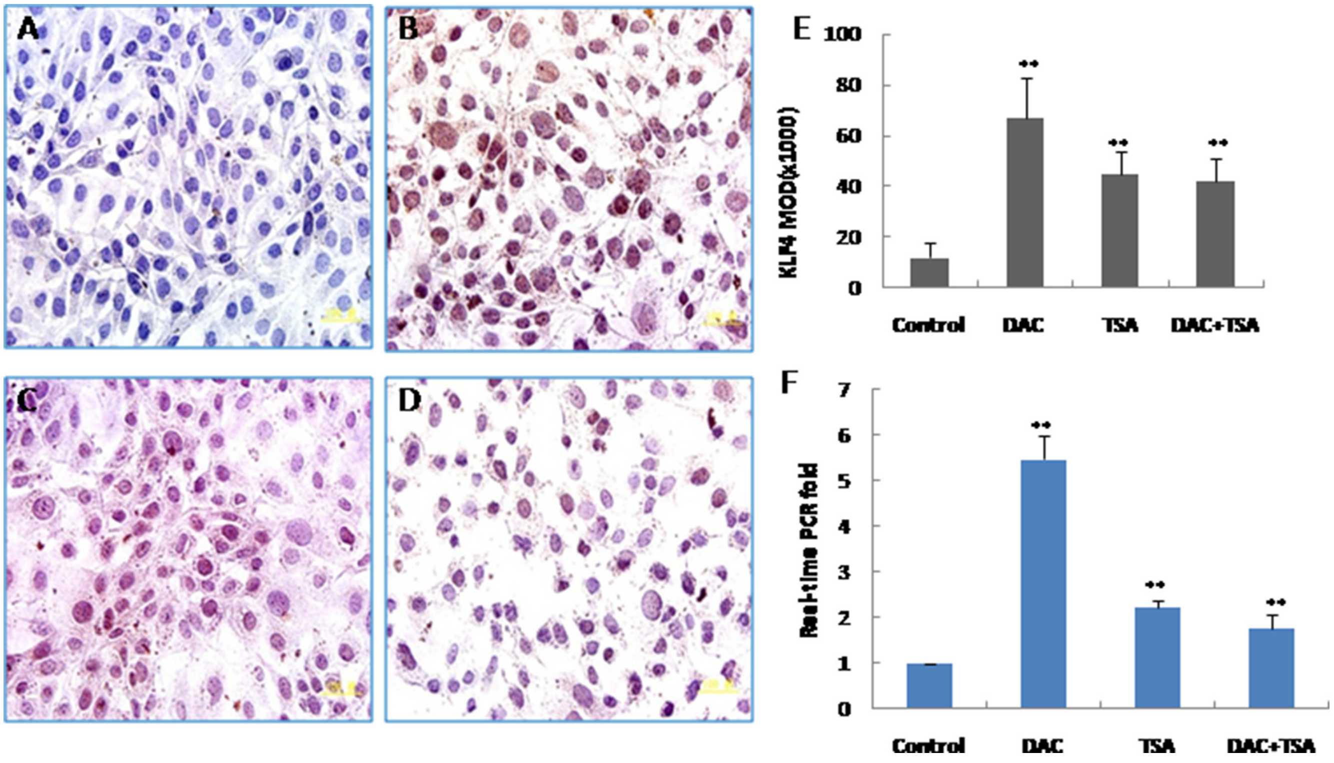

Supplementary Figure S1: KLF4 is hypermethylated in OSCC cell line CAL27. (**A**) Expression of KLF4 in human oral squamous cell carcinoma cell line CAL27 by immunocytochemistry. (**B**) KLF4 expression was increased markedly after treatment with 5 mM DAC for 3 days. (**C**) KLF4 expression was also increased after treatment with 300 nM TSA for 1 day. (**D**) DAC+ TSA treatment. (**E**) KLF4 expression was showed by MOD value. (**F**) KLF4 expression was detected by RT real-time PCR in CAL27 cells after treatment with 5 mM DAC, 300 nM TSA alone and their combination. $^{**}P < 0.01$ as compared with the control group.

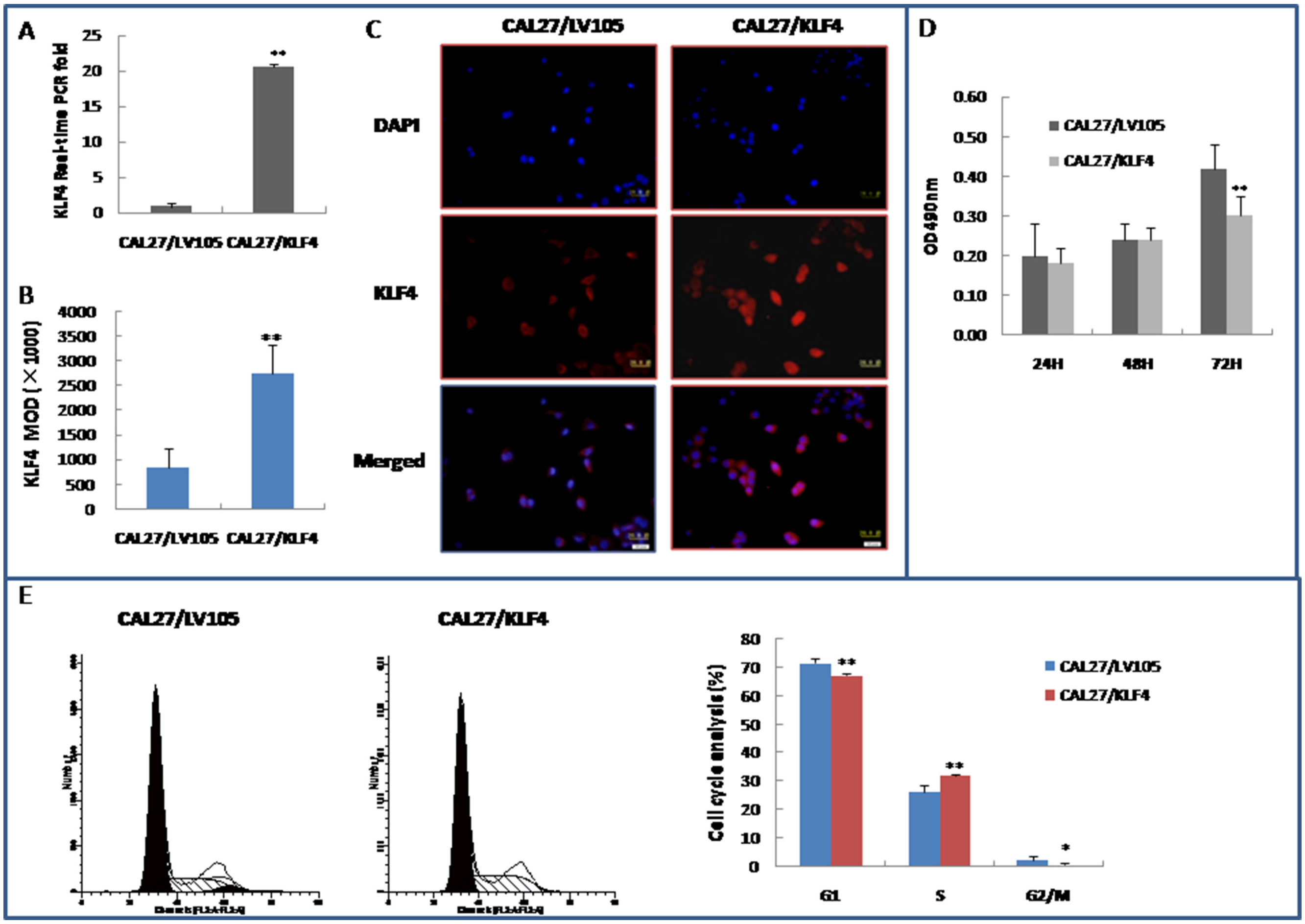

Supplementary Figure S2: Stable transduction of KLF4 into CAL27 cells could slow down cell growth, inhibit cell cycle G2/M phase. (**A**) Stable transduction of lentivirus LV105/KLF4 into CAL27 cells as showed by CAL27/KLF4 (CAL27/LV105 served as control) increased KLF4 expression by RT–PCR. (**B** and **C**) Stable transduction of LV105/KLF4 into CAL27 cells increased KLF4 expression and localized in the nuclei of CAL27 cells by immunocytochemistry. (**D**) SCC15/KLF4 cells grew slower than CAL27/LV105 cells, as determined by a MTT-based assay. (**E**) KLF4 transduction inhibited cell cycle G2/M phase of CAL27 cells by flow cytometry assay. *$P < 0.05$; **$P < 0.01$ as compared with the control group.

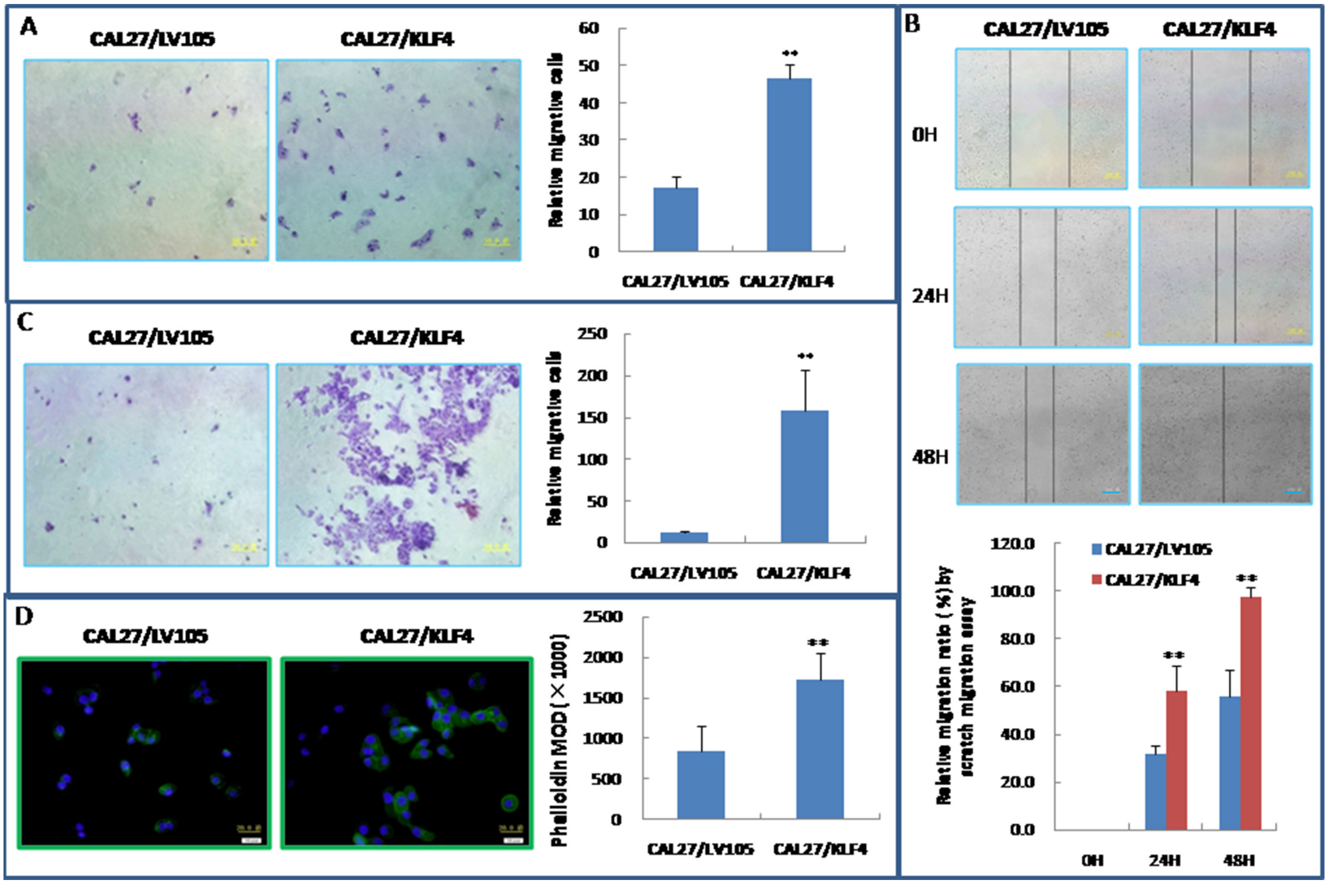

Supplementary Figure S3: Stable transduction of KLF4 into CAL27 cells could increase cell migration and invasion ability. (**A**) KLF4 transduction promoted CAL27 cell migration ability by trans-well migration assay. (**B**) KLF4 transduction promoted CAL27 cell migration ability by scratch migration assay.(**C**) KLF4 transduction promoted CAL27 cell invasive ability by trans-well invasion assay. (**D**). KLF4 transduction increased phalloidin staining in CAL27 cells. $^{**}P < 0.01$ as compared with the control group.

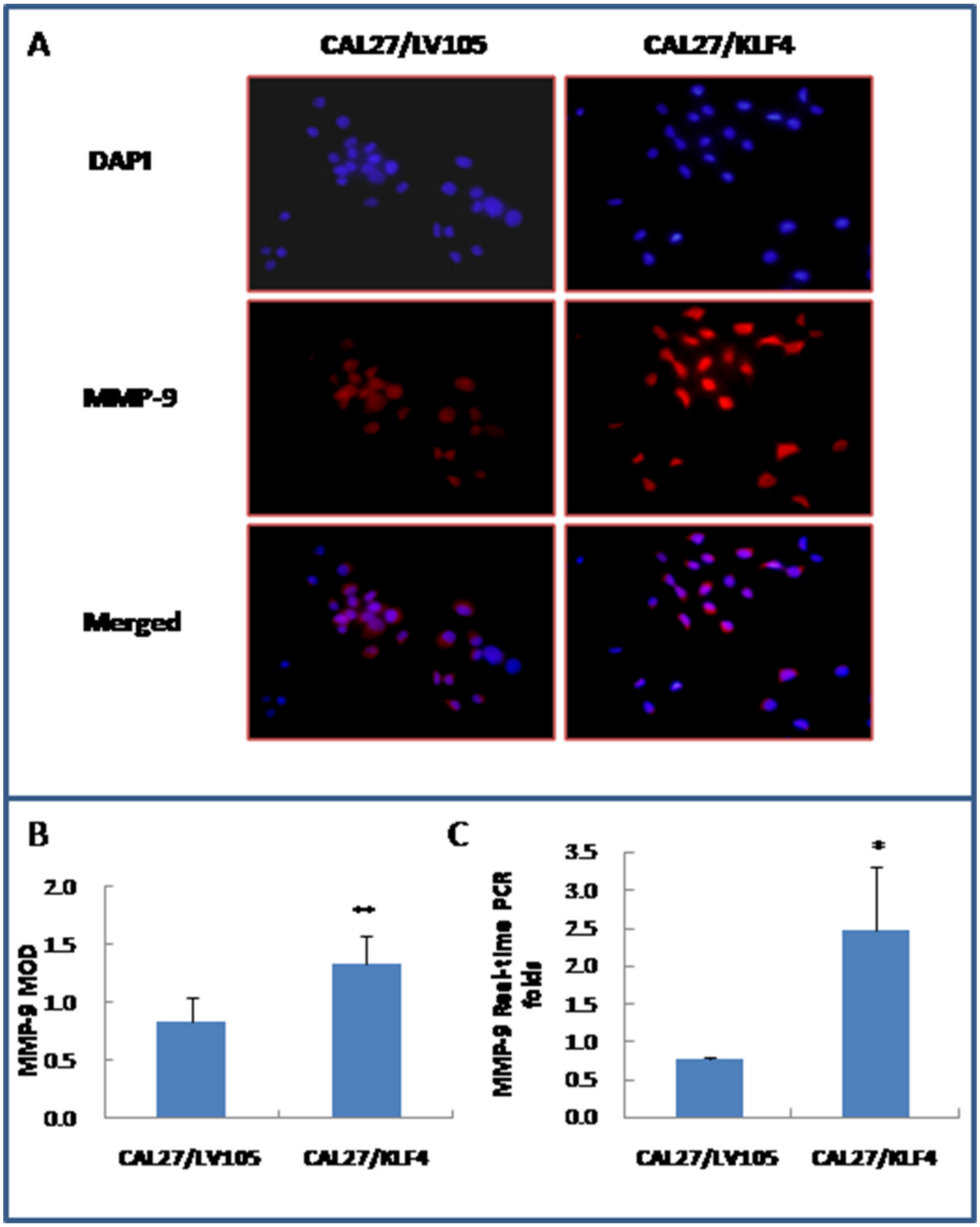

Supplementary Figure S4: Stable transduction of KLF4 into CAL27 cells increased MMP-9 expression. (**A** and **B**) KLF4 transduction increased MMP-9 expression by immunocytochemistry. (**C**) KLF4 transduction increased MMP-9 expression by Real-time PCR detection. *$P < 0.05$; **$P < 0.01$ as compared with the control group.

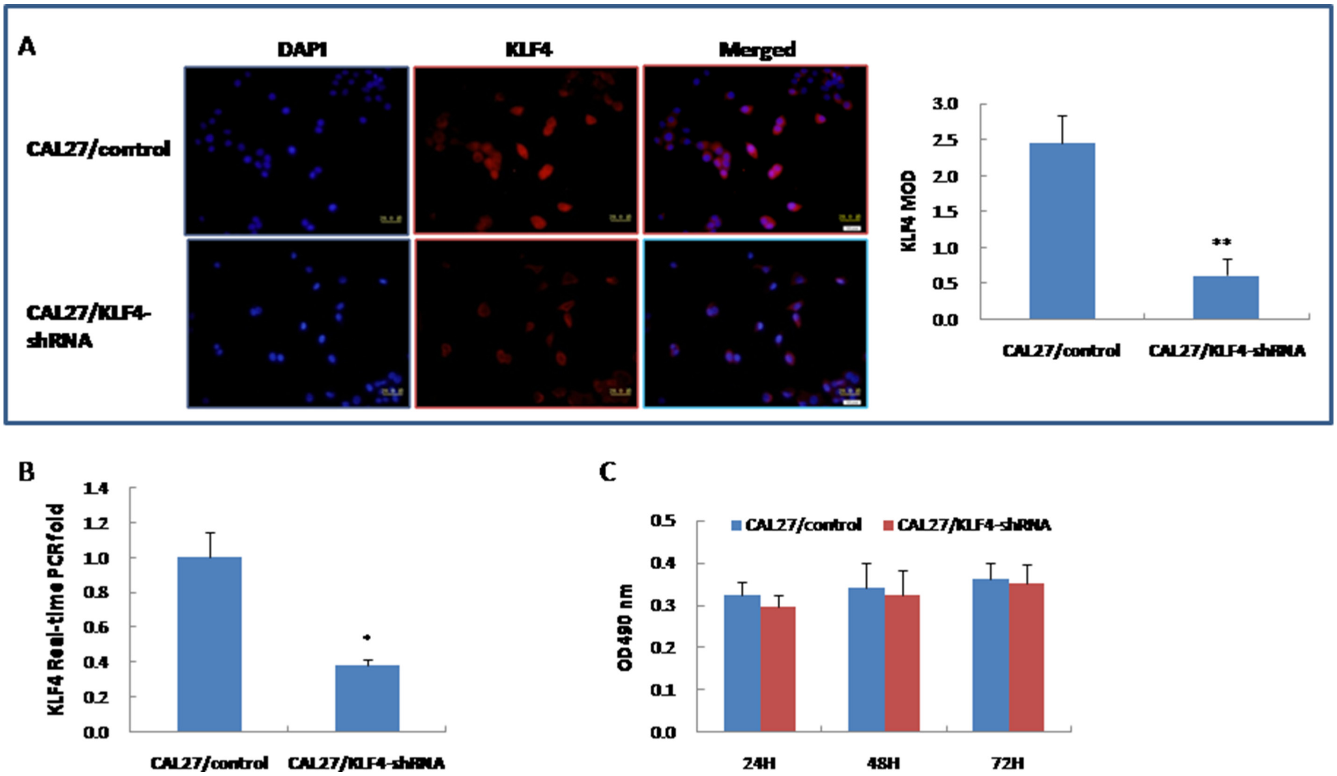

Supplementary Figure S5: Stable transduction of KLF4-shRNA lentivirus into CAL27 cells. (**A**) Stable transduction of lentivirus LV3/KLF4-shRNA into CAL27 cells as showed by CAL27/KLF4-shRNA (CAL27/control served as control) decreased KLF4 expression by immunocytochemistry, (**B**) KLF4 expression by RT–PCR. (**C**) MTT assay. *$P < 0.05$; **$P < 0.01$ as compared with the control group.

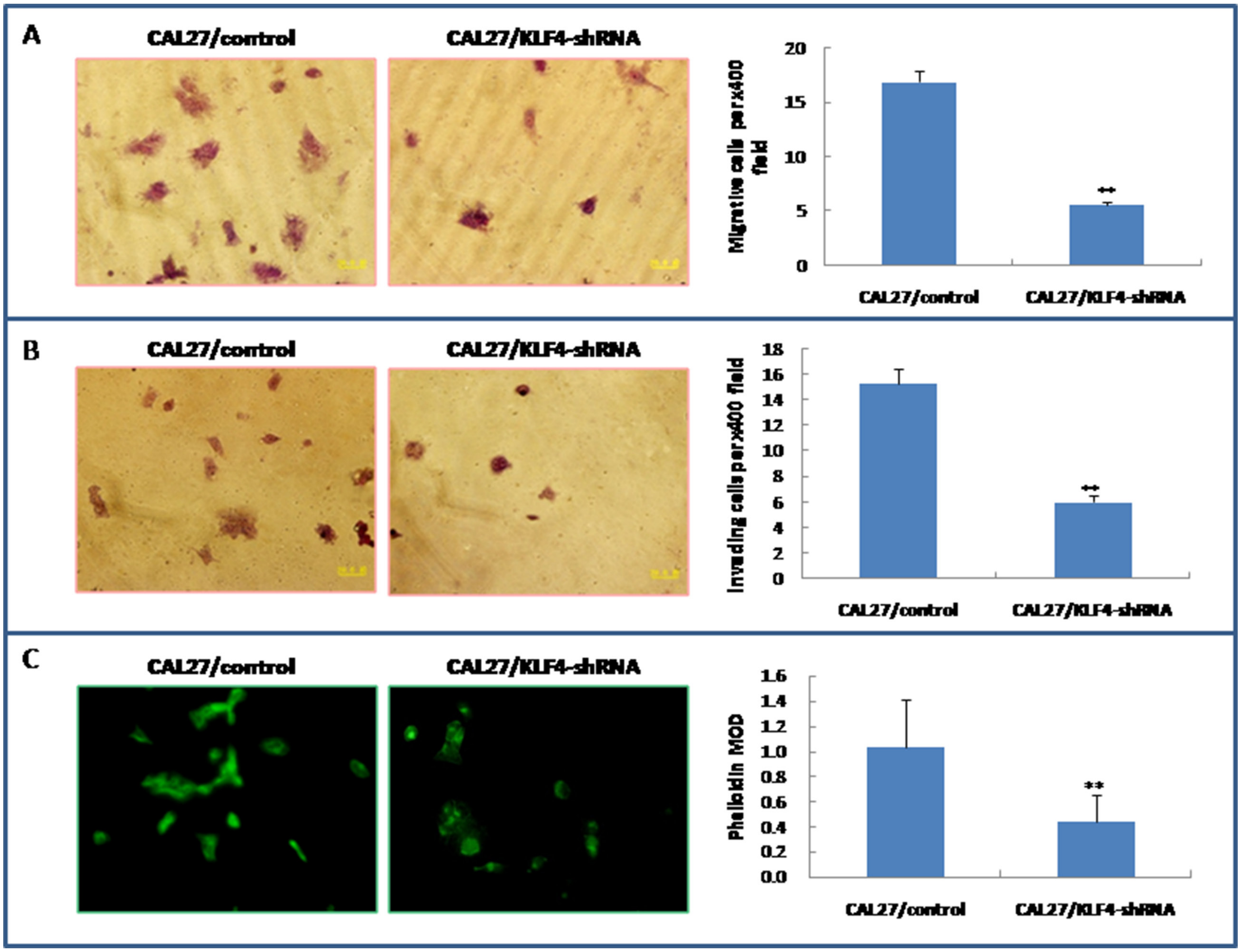

Supplementary Figure S6: Stable transduction of KLF4-shRNA into CAL27 cells could decrease cell migration and invasion ability. (**A**) KLF4-shRNA transduction inhibited CAL27 cell migration ability by trans-well migration assay. (**B**) KLF4-shRNA transduction inhibited CAL27 cell invasive ability by trans-well invasion assay. (**C**) KLF4-shRNA transduction decreased phalloidin staining. *$P < 0.05$; **$P < 0.01$ as compared with the control group.

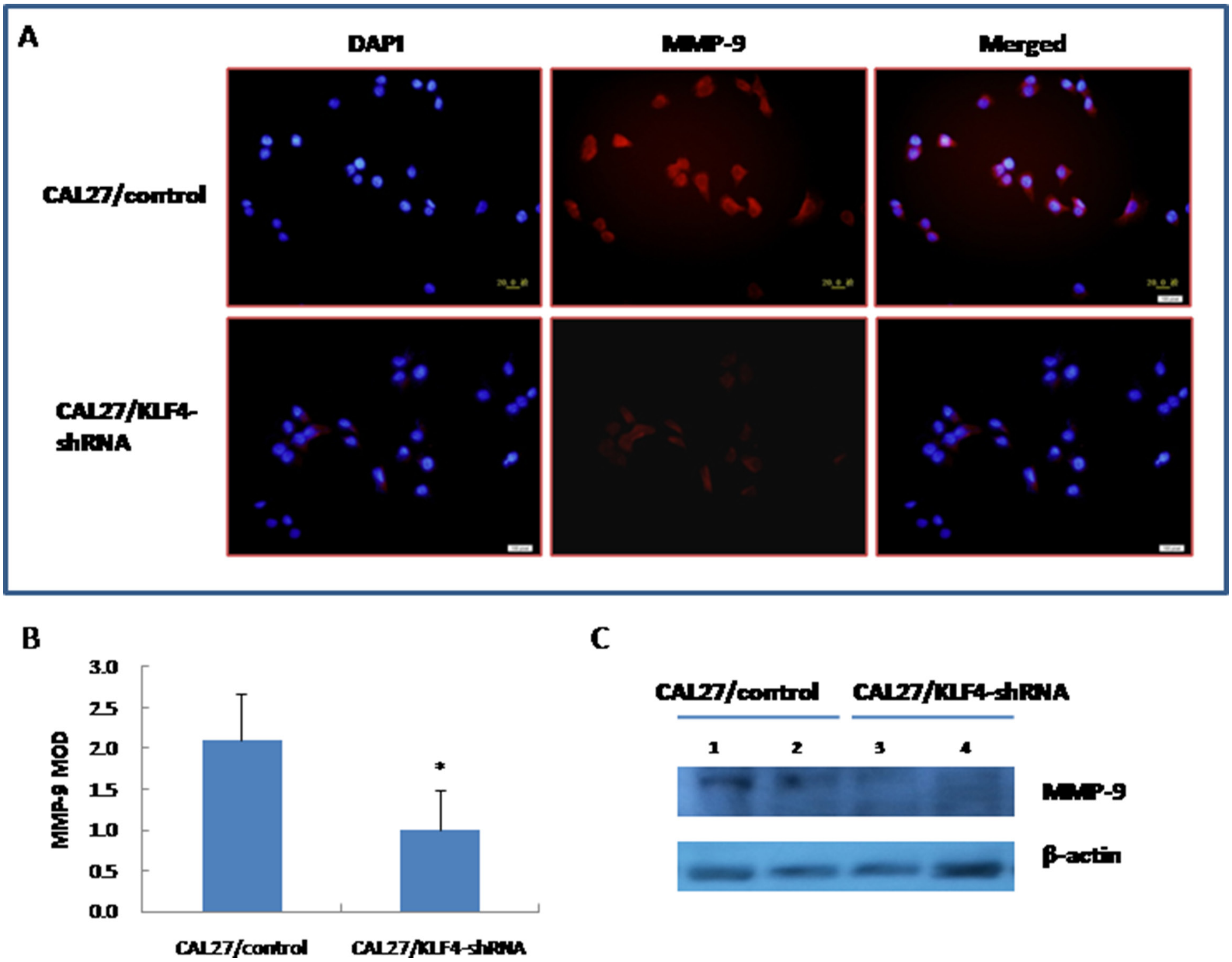

Supplementary Figure S7: Stable transduction of KLF4-shRNA into CAL27 cells decreased MMP-9 expression. (**A** and **B**) KLF4-shRNA transduction decreased MMP-9 expression by immunocytochemistry. (**C**) KLF4-shRNA transduction decreased MMP-9 expression by western blotting. **P* < 0.05 as compared with the control group.

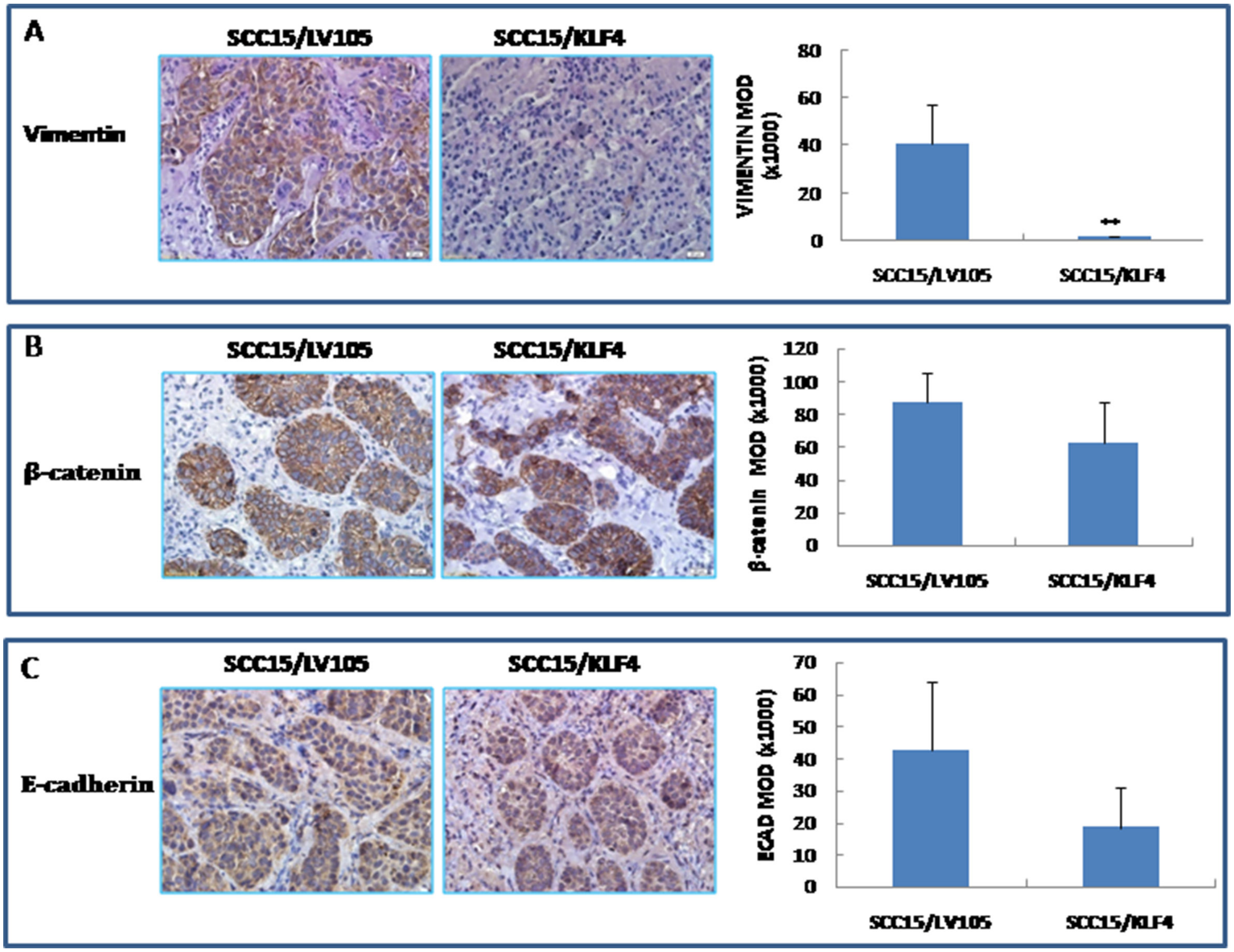

Supplementary Figure S8: (**A**) KLF4 transduction decreased vimentin expression by IHC in SCC15/KLF4 xenografted tumors. ***P* < 0.01 as compared with the control group. (**B**) Expression of β-catenin by IHC in SCC15/KLF4 xenografted tumors. (**C**) Expression of E-cadherin by IHC in SCC15/KLF4 xenografted tumors.
